# One-Pot Preparation of Layered Double Hydroxide-Engineered Boric Acid Root and Application in Wastewater

**DOI:** 10.3390/molecules29133204

**Published:** 2024-07-05

**Authors:** Fengrong Zhang, Cuilan Zhang, Kaixuan Zhang, Lishun Wu, Dandan Han

**Affiliations:** 1 School of Chemistry and Chemical Engineering, Heze University, Heze 274015, China; 15254096837@163.com (K.Z.); 19105407006@163.com (L.W.); danicahan1125@163.com (D.H.); 2 Guiyang Road Primary School, Heze 274015, China; 18754041693@163.com

**Keywords:** layered double hydroxides, heavy metals, methylene blue, adsorption, solid concentration effect, NH_4_B_5_O_8_

## Abstract

Heavy metals and organic pollutants are prevalent in water bodies, causing great damage to the environment and human beings. Hence, it is urgent to develop a kind of adsorbent with good performance. Anion interlacing layered double hydroxides (LDHs) are a promising adsorbent for the sustainable removal of heavy metal ions and dyes from wastewater. Using aluminum chloride, zinc chloride and ammonium pentaborate tetrahydrate (NH_4_B_5_O_8_ · 4H_2_O, BA) as raw materials, the LDHs complex (BA-LDHs) of B_5_O_8_^−^ intercalation was prepared by one-step hydrothermal method. The BA-LDHs samples were characterized by a X-ray powder diffractometer (XRD), scanning electron microscope (SEM), Fourier transform infrared spectrometer (FT-IR) and the Brunauer–Emmett–Teller (BET) method. The results showed that B_5_O_8_^-^ was successfully intercalated. Adsorption experimental results suggested that BA-LDHs possess a maximum adsorption capacity of 18.7, 57.5, 70.2, and 3.12 mg·g^−1^ for Cd(II), Cu(II), Cr(VI) and Methylene blue (MB) at *C*_s_ = 2 g·L^−1^, respectively. The adsorption experiment conforms to the Langmuir and Freundlich adsorption models, and the kinetic adsorption data are well fitted by the pseudo-second-order adsorption kinetic equation. The as-prepared BA-LDHs have potential application prospects in the removal of heavy metals and dyes in wastewater. More importantly, they also provide a strategy for preparing selective adsorbents.

## 1. Introduction

Heavy-metal and organic pollution is a significant environmental issue that has raised global concern due to its harmful impact on humans and the natural ecosystem [1]. Cd(II) and Cu(II), as two typical cationic heavy metals, are common in wastewater. Cd(II) and its compounds were included in the list of toxic and hazardous water pollutants (the first batch), and were also in the list of group 1 carcinogens. Excess Cd(II) in the human body can damage the kidney and liver and lead to osteoporosis and the softening of bones. Japan has reported a chronic cadmium poisoning phenomenon known as “Ita-ita-sickness” caused by the ingestion of cadmium-contaminated water sources. In a food sample, rice and rice products in Hunan were found to contain excessive levels of cadmium. Though Cu(II) is one of the essential trace minerals in the human body, if the body ingests too much, it may also cause great harm to organisms and even endanger aquatic organisms and human lives by binding with enzymes and proteins [2,3]. Hexavalent chromium Cr(VI), like Cd(II), was also listed in the list of toxic and hazardous water pollutants (the first batch), and also in the list of group 1 carcinogens. Common species of Cr(VI) in wastewater are CrO_4_^2−^, H_2_Cr_2_O_7_, Cr_2_O_7_^2−^, and HCrO_4_^−^, which have more toxicity compared with Cr(III) and can be easily absorbed and stored, thereby considerably harming human health [4,5,6]. Cr(VI) can cause kidney and liver damage and has carcinogenic effects. Methylene blue (MB), a commonly used dye, was listed as a group 3 carcinogen by the World Health Organization’s International Agency for Research on Cancer. The harmful components of this kind of dye may be absorbed by the skin and diffuse in the human body after long-term contact, affecting the normal metabolic reaction of the human body [7]. Therefore, the removal of heavy metal ions and dyes from wastewater is of great significance.

Generally, the techniques applied in the treatment of heavy metal ions and dye contamination include chemical precipitation, electro-chemical treatment, ion exchange, membrane filtration, catalysis and the adsorption method. Among these, the chemical precipitation method produces large amount of sludge, which may result in the problem of secondary contamination. Electro-chemical treatment expends a lot of electric energy. Furthermore, the ion-exchange and membrane filtration methods cannot be used at a large scale, owing to their high cost. Of note, catalysis plays a crucial role in many chemical processes, in particular for organic wastes that are hardly degradable through a conventional biodegradation process, such as dyes and textile assistants [8]. However, the adsorption method is an efficient and economical means of removing aqueous heavy metals and dyes [9,10,11,12] because of its convenient maintenance and low cost [13,14] in mono-pollutant and binary polluted systems. It involves attracting the pollutants to the surface of adsorbents via coordination bonds, π-π interaction, hydrogen bonds and electrostatic attraction.

Nanomaterials, used as adsorbents to remove heavy metal ions and dyes from wastewater, have received significant attention owing to their high specific surface area. As a nanomaterial, hydrotalcites (also known as layered double hydroxides, LDHs) are a class of anionic layered clays whose chemical composition is [M^2+^_1−x_ M^3+^_x_ (OH)_2_]^x+^[A^n−^]_x/n_·mH_2_O, of which M^2+^ is a bivalent metal ion, such as Mg^2+^, Zn^2+^, Cu^2+^, Co^2+^, and Ni^2+^; M^3+^ is a trivalent metal ion, such as Al^3+^, Fe^3+^, and Cr^3+^; and A^n-^ is an interlayer anion, such as NO_3_^−^, CO_3_^2−^, and Cl^−^ [15]. The cationic structure layer and interlayer exchangeable ions are connected by non-covalent bonds such as static gravity and hydrogen bonds, mainly hydrogen bonds with weak binding force, among which x is generally between 0.2 and 0.8. Interlayer anions and bound water molecules of hydrotalcite can be exchanged or removed without destroying their own layered structures. The special structure of LDHs gives them the controllability of layer cations, controllability of interlayer anions, and thermal stability, and they are also flame retardant. At the same time, LDHs have been studied and applied more and more widely due to their special properties, and the main applications now include adsorbents, catalysts, ion-exchange carriers, pharmaceutical production, cement additives, wastewater treatment and other industries and fields [16,17,18,19].

Boron is an electron-deficient element with strong oxyphilic property and has the characteristics of inorganic polymer elements. It exists mainly in the form of borate in nature. In borate, boron atoms form planar triangular (BO_3_) units with sp^2^ hybridization, or tetrahedral (BO_4_) units with sp^3^ hybridization, which can be linked to form mononuclear- or multinuclear-containing boron and oxygen anions. Borates are listed as critical materials, main sources of boron, and have a wide range of industrial applications [20]. Wang et al. reported a study on the effective removal of Cr(VI) by highly crystalline aluminum borate BAC(10), which was hydrothermally synthesized from a mixture of boric acid, anhydrous aluminum trichloride, and ammonium hydroxide at 150 °C for 18 h [21]. However, ammonium hydroxide has the characteristics of volatility and corrosion, and it is limited in the process of use. Most of the LDHs of boric acid intercalation were prepared by the ion-exchange method [22].

This study aims to prepare a new adsorbent through a simple method to improve the adsorption efficiency of NH_4_B_5_O_8_-modified LDHs for pollutants. Of late, the preparation of LDHs from NH_4_B_5_O_8_ has rarely been reported. Herein, a composite adsorbent (BA-LDHs) was developed using a facile and convenient one-pot synthesis method and then adopted for Cd(II), Cu(II), Cr(VI) and MB adsorption. By characterizing the BA-LDHs and comparing the adsorption performance for different pollutants containing heavy-metal cations (Cd(II), Cu(II)) and anions (Cr(VI)) and dyes (MB), the investigation included the following steps: (1) prepare and characterize BA-LDHs; (2) investigate the removal behaviors of Cd(II), Cu(II), Cr(VI) and MB by this new sorbent; and (3) clarify the mechanisms involved in Cd(II), Cu(II), Cr(VI) and MB adsorption. BA-LDHs are an economical and practicable material for removing heavy metals and dyes from water and provide a reference for sewage treatment.

## 2. Results and Discussion

### 2.1. Characterization of Adsorbents

**Figure 1:**
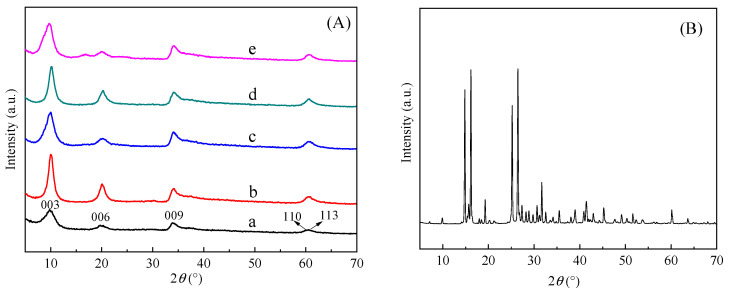
Experimental XRD patterns of (**A**): (a) BA-LDHs, (b) BA-LDHs-Cd, (c) BA-LDHs-Cu, (d) BA-LDHs-Cr and (e) BA-LDHs-MB; (**B**): BA.

Figure 1 shows the powder X-ray diffraction (XRD) patterns of all samples in the 2*θ* range of 5–70°. It is well known that the XRD peak intensity depends on the number of diffraction planes of the incident X-ray quantum at the same 2*θ* values. The interlayer spacing of the LDH layers was calculated according to Bragg’s equation. Observing the XRD spectra of BA-LDH, the primary characteristic peaks corresponded to 2*θ*~9.99°, 20.1°, 33.9°, 60.3° and 61.2°, namely 003, 006, 009, 110 and 113, respectively, which was closely associated with the lamellar structure of LDH. This observation implies the successful preparation of ZnAl-LDH, which was consistent with the crystal ZnAl-LDHs reported in the literature [23,24,25]. The corresponding interlayer spacing values at 003, 006 and 009 are 0.89 nm, 0.44 nm and 0.26 nm, respectively. As can be seen, the intensity of the characteristic diffraction peaks of BA decreases in the BA-LDHs, which means that BA was involved in the reaction. The size of B_5_O_8_^−^ was calculated by Gauss as 0.86 nm × 0.59 nm × 0.18 nm, which means the pentaborate ions (B_5_O_8_^−^) may enter the LDHs interlayer. Except for the increase in peak intensity, no other characteristic peaks were detected in BA-LDHs-Cd, BA-LDHs-Cu, BA-LDHs Cr and BA-LDHs-MB. So, the material consisted of these two phases. This indicates that the adsorbed samples retained their original crystal structures very well.

**Figure 2:**
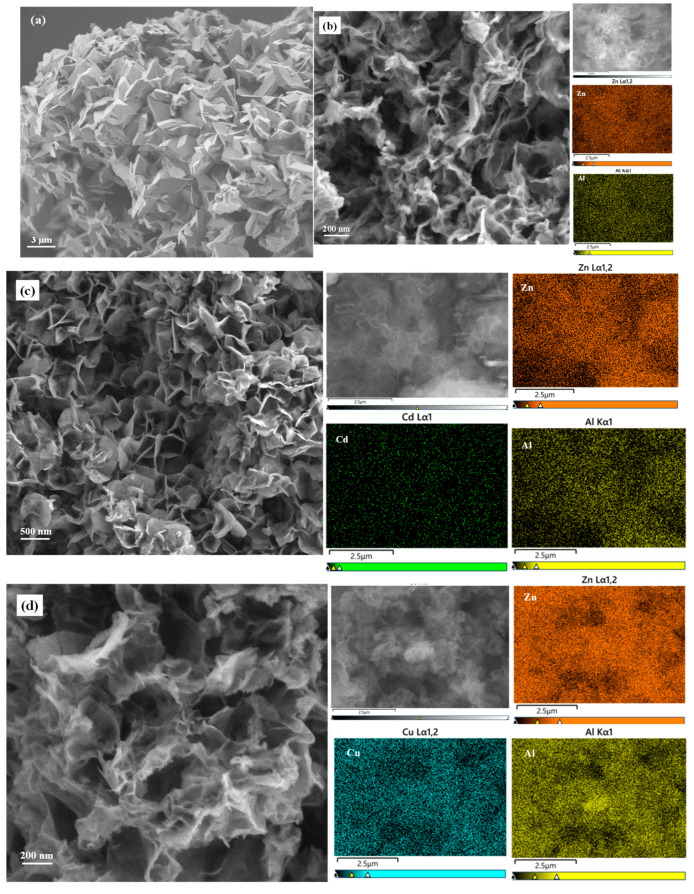

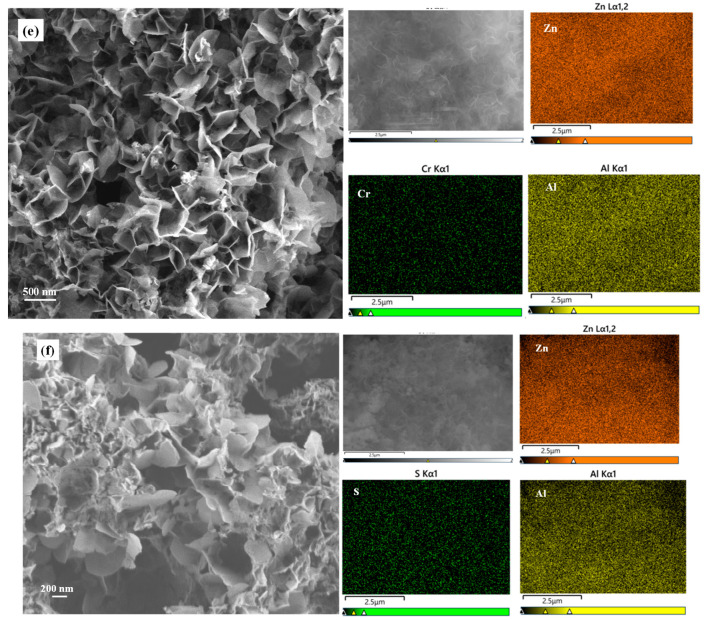
SEM images and corresponding elements distribution mapping of. (**a**) NH_4_B_5_O_8_ · 4H_2_O; (**b**) BA-LDHs; (**c**) BA-LDHs-Cd; (**d**) BA-LDHs-Cu; (**e**) BA-LDHs-Cr; (**f**) BA-LDHs-MB.

The SEM image of NH_4_B_5_O_8_ · 4H_2_O (Figure 2a) presents the morphology of a rhombic system with two vertebrae. When NH_4_B_5_O_8_ · 4H_2_O was heated above 90 °C, it decomposed and released ammonia, which provided an alkaline environment when preparing BA-LDHs, without adding additional alkaline substances. This method of preparing BA-LDHs was similar to urea hydrolysis, which has rarely been reported. Before adsorption, BA-LDHs’ appearance was composed of many small flakes piled up (Figure 2b). However, after the adsorption of Cd(II), Cu(II), Cr(VI) and MB, the morphology of samples changed significantly. The BA-LDHs-Cd and BA-LDHs-Cu showed that the pieces were connected to each other, with loose porous and folded states (Figure 2c,d). The morphology of BA-LDHs-Cr and BA-LDHs-MB had some large sheet structures (Figure 2e,f). The above results were consistent with those of XRD characterization (Figure 1). In the element distribution mapping of the adsorbed samples, it can be seen that Cd(II), Cu(II), Cr(VI) and S (the S element was the MB containing a detection marker) are detected, respectively. Moreover, the surface area of the BA-LDHs was 101.31 m^2^·g^−1^. This was agreed with the reported ZnAl-LDH-BC_600_ (102.56 m^2^·g^−1^), which was from the ZnAl-LDHs modified by biochar at 600 °C [26]. The pore size (*D*_p_) and pore volume (*V*_p_) of BA-LDHs were 6.67 nm and 0.152 cm^3^·g^−1^, respectively. This demonstrated that the above structure of BA-LDHs could provide a good adsorption site.

**Figure 3:**
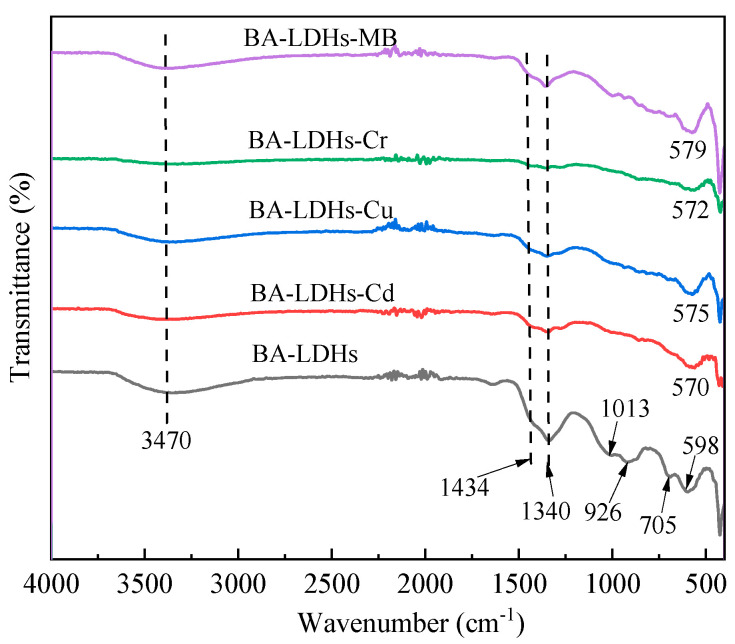
Fourier transform infrared spectroscopy (FT-IR) spectra of samples before and after Cd(II), Cu(II), Cr(VI) and MB adsorption.

The FT-IR spectra for BA-LDHs, BA-LDHs-Cd, BA-LDHs-Cu, BA-LDHs-Cr and BA-LDHs-MB are presented in Figure 3. There is an intense band around 3470 cm^−1^ in all samples, which corresponds to the stretching of the hydroxyl bond (O-H), relative to both the hydration water of the interlayer space and the hydroxyl group present in the LDH layers [23]. The band at 1340 cm^−1^ was assigned to the stretching of CO_3_^2−^ in the samples, which was due to dissolved CO_2_ in the aqueous medium [27]. The stretching vibration of the B-O group manifested as a shoulder peak at 1434 cm^−1^ [23]. Additionally, the vibration signals at 1013, 926, 705 and 598 cm^−1^ could be attributed to M–O (Zn-O and Al-O) [23]. Moreover, the vibrational signals at 598 cm^−1^ in BA-LDHs and the phenomenon of redshift, which occurred in all samples after adsorption, indicated that the adsorption mechanism was not consistent in each adsorption system (see Section 3.4. Adsorption mechanism).

### 2.2. Adsorption Kinetics

**Figure 4:**
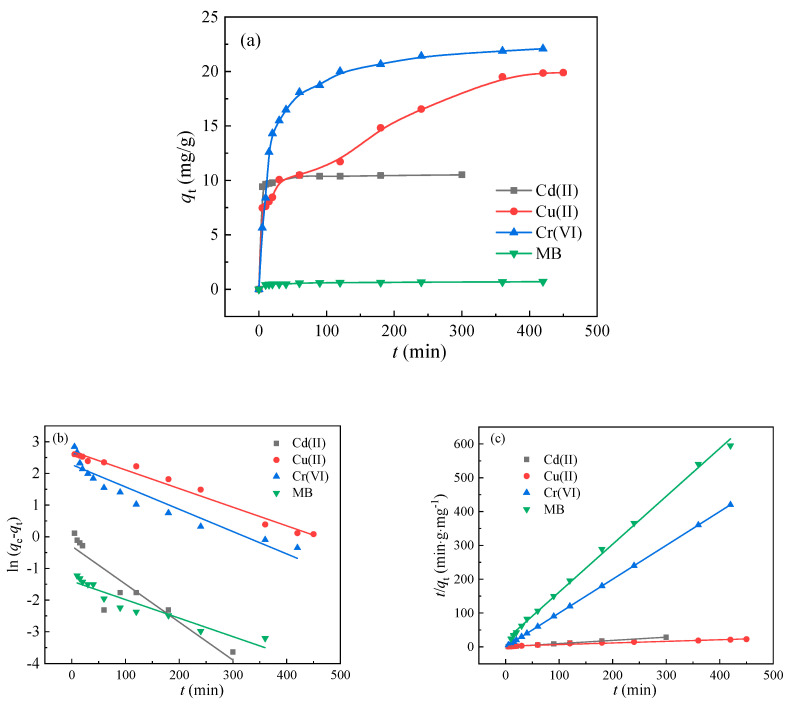
(**a**) Kinetics, (**b**) pseudo-first-order kinetics and (**c**) pseudo-second-order kinetics for Cd(II), Cu(II), Cr(VI) and MB sorption on BA-LDHs samples.

The adsorption amount and adsorption speed of the adsorbents on the pollutants could be deduced by kinetic behavior, so as to determine the underlying mechanism. The adsorption kinetics of BA-LDHs to Cd(II), Cu(II), Cr(VI) and MB are shown in Figure 4a. As can be seen from the adsorption kinetics curves of BA-LDHs for Cd(II) and MB, the adsorption equilibrium could be quickly reached within 30 min. However, the adsorption of BA-LDHs for Cu(II) and Cr(VI) comprised two phases of fast adsorption and slow adsorption. At the beginning of the 100 min, the adsorption capacity increased sharply with the increase in adsorption time and then gradually slowed down and reached saturation. The reason for this phenomenon could be attributed to an abundance of unoccupied active sites on the BA-LDHs at the beginning of the reaction [28], whereas as the reaction continued, the majority of the sites on BA-LDHs were occupied by Cu(II) and Cr(VI), until they were filled [29].

(Cd(II): *C*_0_ = 200 mg·L^−1^, Cu(II): *C*_0_ = 100 mg·L^−1^, Cr(VI): *C*_0_ = 100 mg·L^−1^,

MB: *C*_0_ = 4 mg·L^−1^, 25 °C, *C*_s_ = 4 g·L^−1^)

For a better elucidation of the adsorption mechanism, quasi-first-order and quasi-second-order rate equations were used to fit the experimental data, respectively (Figure 4b,c). Table 1 lists the fitted rate equation parameters and linear correlation coefficient (*R*^2^) values. By comparison, the quasi-second-order rate equation can better describe the experimental results.

### 2.3. Adsorption Isotherms

**Figure 5:**
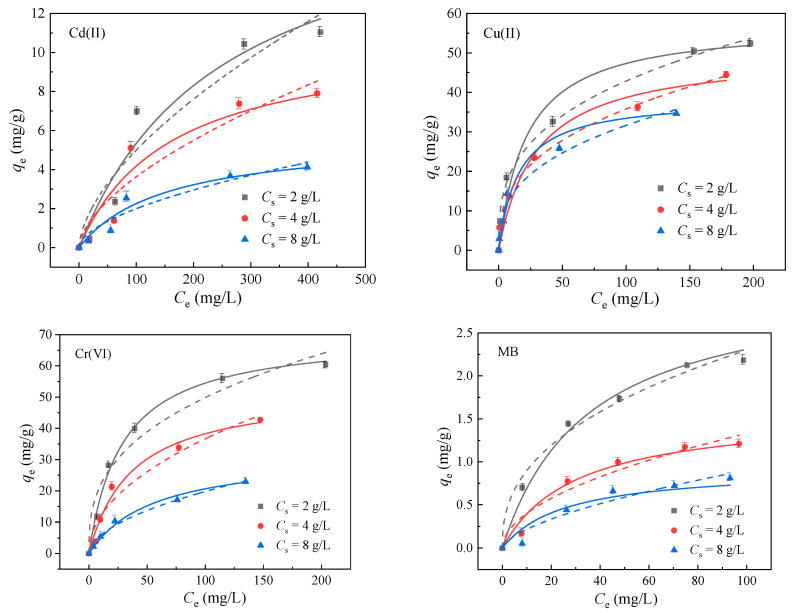
Sorption isotherms of Cd(II), Cu(II), Cr(VI) and MB adsorption onto BA-LDHs at different sorbent dosages. (25 °C). The dots represent experimental data, the solid lines represent Langmuir model fits, and the dashed lines represent Freundlich model fits.

An adsorption isotherm is an important tool to obtain the information about the properties of adsorption layers and the interaction between adsorbed and adsorbed substances [30]. Langmuir and Freundlich, as broadly accepted isotherm models, were introduced in the present study to elaborate the adsorption behavior of Cd(II), Cu(II), Cr(VI) and MB onto BA-LDHs. The Langmuir model is based on the premise that monolayer adsorption occurs on a uniform surface, and it could be used to predict the maximum adsorption capacity of the adsorbent [31]. According to the Freundlich model, the surface of the adsorbent is heterogeneous, and the adsorbent is adsorbed in multiple layers [32]. The Langmuir and Freundlich isotherms were used for the nonlinear fitting of the above adsorption equilibrium data, respectively. The results showed that both Langmuir and Freundlich isotherms could describe the adsorption isotherms of the adsorption system under each *C*_s_ (Figure 5). Table 2 lists the fitting values of the model parameters by Langmuir and Freundlich isothermal equations. It could be seen that the saturation adsorption *q*_m_ decreases with the increase in *C*_s_, which was known as the solid concentration effect [33,34,35]. It was found that other parameters also change with the change of *C*_s_. However, neither the Langmuir nor Freundlich isotherm could describe or predict this phenomenon.

### 2.4. Adsorption Mechanism

**Figure 6:**
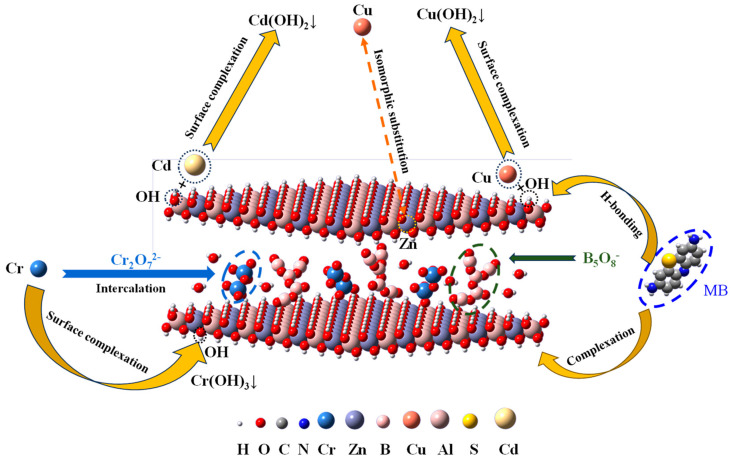
Proposed mechanism schematic.

Overall, taking into account the characterization analysis and batch adsorption experimental results, the adsorptions of Cd(II), Cu(II), Cr(VI) and MB on BA-LDHs were complex process concerning several specific mechanisms. The apparently optimized porosity structure and expanded surface area (BET and SEM) of BA-LDHs yielded an abundance of available sorption sites for Cd(II), Cu(II), Cr(VI) and MB to be filled in the pores channel. The Freundlich isotherm model matched experimental data well, illustrating that the surface sites of BA-LDHs were heterogeneous, and its entrapment of Cd(II), Cu(II), Cr(VI) and MB could be driven by multilayer physical adsorption. According to the saturated adsorption capacity (*q*_m_) of BA-LDHs for pollutants, the adsorption capacity for each pollutant is Cr(VI) > Cu(II) > Cd(II) > MB at *C*_s_ = 2 g·L^−1^ (Table 2). Briefly, the main mechanism of Cr(VI) removal from water was tied to the isomorphic substitution of Cr^6+^ and Zn^2+^ and the intercalation of Cr_2_O_7_^2−^, except Cr(OH)_3_ precipitation. The former is because the radius of Cr^6+^ (0.044 nm) is smaller than the radius of Zn^2+^ (0.074 nm), and the latter is because the successful intercalation of B_5_O_8_^-^ (0.86 nm × 0.59 nm × 0.18 nm) in the early stage opens a channel for Cr_2_O_7_^2−^ (0.55 nm × 0.25 nm × 0.21 nm) to enter. The main mechanism of removal of Cu(II) from water is isomorphic substitution, except Cu(OH)_2_ precipitation, mainly because the ionic radius of Cu(II) (0.073 nm) is very close to that of Zn^2+^ (0.074 nm). The main mechanism of Cd(II) removal from water is Cd(OH)_2_ precipitation on the surface of the adsorbent. The main mechanism of MB removal was on account of H-bonding and complexation. A suggested reaction pathway for the adsorption is presented in Figure 6.

## 3. Materials and Methods

### 3.1. Chemicals and Materials

The reagents required for the current work were of analytical grade. Aluminum chloride hexahydrate (AlCl_3_ · 6H_2_O), zinc chloride (ZnCl_2_) and cadmium pellets were purchased from Tianjin Kemi Ou Chemical Reagent Co., Ltd., Tianjin, China. Nitric acid (HNO_3_), cupric nitrate (Cu(NO_3_)_2_) and potassium dichromate (K_2_Cr_2_O_7_) were purchased from Sinopharm Chemical Reagent Co., Ltd., Shanghai, China. Urea was purchased from Shanghai Aladdin Biochemical Technology Co., Ltd., Shanghai, China. Pentaborate amine tetrahydrate (NH_4_B_5_O_8_ · 4H_2_O) was purchased from Shanghai Maclin Biochemical Technology Co., Ltd., Shanghai, China. Water was purified with a Hitech-Kflow water purification system (Hitech, Beijing, China) (DW).

### 3.2. Fabrication of BA-LDHs Composites

A composite adsorbent (BA-LDHs) was manufactured by a one-pot hydrolysis method with ZnCl_2_, AlCl_3_ · 6H_2_O and boric acid root (B_5_O_8_^−^). Specifically, first, 0.4080 g of ZnCl_2_, 0.2414 g of AlCl_3_ · 6H_2_O with a Zn/Al molar ratio of 3:1, and 2.720 g of NH_4_B_5_O_8_ · 4H_2_O (BA) were mixed by a digital-display constant-temperature magnetic stirrer (XMTD-204, Jintan city Jiangnan Instrument Factory, Jinan, China) thoroughly in 60 mL aqueous solution for 30 min to obtain a homogeneous suspension. Next, the aqueous dispersion obtained above was transferred to in a 100 mL Teflon-lined stainless-steel reactor for 24 h at 120 °C. The reaction solution was centrifuged at 7500 rpm using a centrifuge (TG16-WS, Changsha Xiangzhi centrifuge Instrument Co., Ltd., Changsha, China). The obtained sediment was washed with DW repeatedly until the pH of the solution remained near 7. Then, the obtained samples were dried at 70 °C for 12 h in an electric thermostatic oven (DHG-9070A, Shanghai Haozhuang Instrument Co., Ltd., Shanghai, China). Finally, the product obtained after grinding using an agate mortar was denoted as BA-LDHs.

### 3.3. Characteristics of Adsorbents

The crystalline mineral ingredients of BA-LDHs and samples after adsorption were determined by powder X-ray diffraction (XRD, D/max-rA, Bruker AXS, Co., Ltd., Karlsruhe, Germany) at 40 kV and 40 mA in the 2*θ* range of 1–10° at a scanning rate of 1°/min and 10–70° at a scanning rate of 10°/min, respectively. The analysis area was more than 2 mm × 2 mm. The morphologies of BA-LDHs and LDHs samples were analyzed using GeminiSEM 300 scanning electron microscopy (Carl Zeiss (Shanghai) Management Co., Ltd., Oberkochen, Germany) under the conditions of 1 kV acceleration voltage, coated with gold-palladium. The specific surface area (*A*_s_) and pore volume (*V*_p_) of the samples were calculated using the Brunauer–Emmett–Teller (BET) and Barrett–Joyner–Halenda (BJH) methods, respectively. The surface functional groups of BA-LDHs and samples after adsorption were determined via a Fourier transform infrared spectrum (FTIR, Nicolet 5700, Thermo, Waltham, MA, USA) in the range of 500–3900 cm^−1^ with a resolution of 4 cm^−1^, and scanning was performed in parallel 256 times.

### 3.4. Adsorption Experiments

The adsorption behavior and mechanisms of the adsorbents with the best sorption capacity for Cd(II), Cu(II), Cr(VI) and MB were then investigated through adsorption kinetics and isotherms.

The Cd(II), Cu(II), Cr(VI) and MB removal tests were carried out at room temperature. The initial concentrations of Cd(II) from 20 to 450 mg·L^−1^ were prepared by dissolving cadmium pellets in nitric acid and deionized water; initial concentrations of Cu(II) from 10 to 280 mg·L^−1^ were prepared by dissolving Cu(NO_3_)_2_ in deionized water; initial concentrations of Cr(VI) from 20 to 300 mg·L^−1^ were prepared by dissolving K_2_Cr_2_O_7_ in deionized water; and initial concentrations of MB from 3.5 to 70 mg·L^−1^ were prepared by dissolving methylene blue in deionized water. In all the above solutions, 0.010 M of NaNO_3_ was applied as maintainer for the constant ionic strength of the solutions. With the usage of 0.1 M HNO_3_ and NaOH solutions, the pH values of the Cd(II), Cu(II), Cr(VI) and MB solutions were adjusted to 5.5.

Adsorption kinetics provided important guidance to estimate the possible adsorption mechanisms. Briefly, the BA-LDHs was accurately weighed (*C*_s_ = 4 g·L^−1^) in different beakers containing Cd(II): *C*_0_ = 200 mg·L^−1^, Cu(II): *C*_0_ = 100 mg·L^−1^, Cr(VI): *C*_0_ = 100 mg·L^−1^, and MB: *C*_0_ = 4 mg·L^−1^ solutions, respectively. Then, the solution was oscillated at 150 r/min at room temperature and sampled at different times (0–450 min). Then, we took an appropriate amount of the solution through a 0.45 μm filter membrane and tested the concentration of Cd(II) and Cu(II) in the filtrate using an atomic absorption spectrophotometer (AAS) (AAS-3600, Shanghai Metash Instruments Co., Ltd., Shanghai, China) equipped with an air-acetylene flame, and Cr(VI) and MB using a visible spectrophotometer (N-5600PC, Shanghai Yuan Analysis Instrument Co., Ltd., Shanghai, China). The experimental error was determined by conducting parallel experiments. Information about the kinetic models, including the pseudo-first-order kinetic model and pseudo-second-order kinetic model, was used to fit the experimental data (see Figure 4 and Table 1).

The adsorption isotherm model could provide important guidance for the adsorption of heavy metals and dyes in solution. Briefly, a certain amount of BA-LDHs was weighed and put into a polyethylene tube containing a certain volume of the above solution: Cd(II) from 20 to 450 mg·L^−1^; Cu(II) from 10 to 280 mg·L^−1^; Cr(VI) from 20 to 300 mg·L^−1^; and MB from 3.5 to 70 mg·L^−1^. The adsorbent dosages were kept at 2 g·L^−1^, 4 g·L^−1^ and 8 g·L^−1^ in different solutions. Then, the above solutions were oscillated at 150 r/min on a thermostatic oscillator until adsorption reached equilibrium. Langmuir and Freundlich isotherm models were introduced to elaborate the adsorption behavior of Cd(II), Cu(II), Cr(VI) and MB onto BA-LDHs (see Figure 5 and Table 2).

Moreover, all tests in the present work were executed in triplicate, and the relative error was less than 4.8%.

The adsorption capacity (*q*_t_ or *q*_e_ (mg·g^−1^)) of adsorbents for the target pollutants (Cd(II), Cu(II), Cr(VI) and MB) was computed according to the following Formulas (1) and (2).

*q*_t_ = (*C*_0_ − *C*_t_)/*C*_s_ (1)

*q*_e_ = (*C*_0_ − *C*_e_)/*C*_s_ (2)

The *C*_0_, *C*_t_, *C*_s_, and *C*_e_ parameters denote initial concentration, concentration of the solution at time *t*, adsorbent dosage, and the concentration of solution at the adsorption equilibrium, respectively.

The samples after Cu(II),Cd(II), Cr(VI) and MB adsorption by BA-LDHs were denoted as BA-LDHs-Cu, BA-LDHs-Cd, BA-LDHs-Cr and BA-LDHs-MB, respectively.

## 4. Conclusions

A BA-LDH composite was manufactured by a facile one-pot pyrolysis approach with ZnCl_2_, AlCl_3_ · 6H_2_O and boric acid root (B_5_O_8_^−^). The BA-LDH samples were characterized by XRD, SEM, FT-IR and BET. The results showed that borate was successfully intercalated. And it exhibited Cd(II), Cu(II), Cr(VI) and MB adsorption ability. Adsorption experimental results suggested that BA-LDHs possess a maximum adsorption capacity of 18.7, 57.5, 70.2, and 3.12 mg·g^−1^ for Cd(II), Cu(II), Cr(VI) and MB at *C*_s_ = 2 g·L^−1^, respectively. The kinetic adsorption data were well fitted by the pseudo-second-order adsorption kinetic equation. The adsorption isotherms conformed to the Langmuir and Freundlich adsorption models. The main mechanism of Cd(II), Cr(VI) and MB removal from water were tied to isomorphic substitution and the intercalation of Cr_2_O_7_^2−^, except Cr(OH)_3_ precipitation; Cu(II) was owing to isomorphic substitution, except Cu(OH)_2_ precipitation; Cd(II) removal was due to Cd(OH)_2_ precipitation on the surface of the adsorbent; and MB removal was due to H-bonding and complexation. The as-prepared BA-LDHs have potential application prospects in the removal of heavy metals and dyes in wastewater. More importantly, this also provides a strategy for preparing selective adsorbents.

## Figures and Tables

**Table 1:** Kinetic model parameters for Cd(II), Cu(II), Cr(VI) and MB adsorption onto BA-LDHs samples (*C*_s_ = 4 g·L^−1^).

Adsorption Object	Pseudo-First-Order: ln(*q*_e_ − *q*_t_) = ln*q*_e_ − *k*_1_t			Pseudo-Second-Order: t/*q*_t_ = 1/*q*_e_^2^*k*_2_ + t/*q*_e_		
	*q*_e_	*k*_1_	*R* ^2^	*q*_e_	*k*_2_	*R* ^2^
Cd	0.7421	0.01199	0.8003	10.54	0.08180	0.9999
Cu	14.85	0.005880	0.9794	20.98	0.001189	0.9736
Cr	9.746	0.007050	0.8955	22.79	0.002853	0.9997
MB	0.2463	0.005840	0.8946	0.7063	0.09844	0.9974

**Table 2:** Nonlinear-fit data of model parameters for Cd(II), Cu(II), Cr(VI) and MB sorption on BA-LDHs at different *C*_s_.

Adsorption Object	*C*_s_ (g·L^−1^)	Langmuir Isotherm: *q*_e_ = *K*_L_q_m_*C*_e_/(1 + *q*_m_*K*_L_)			Freundlich Isotherm: *q*_e_ = *K*_F_*C*_e_*^n^*_F_		
		*q*_m_ (mg·g^−1^)	*K*_L_ (L·mg^−1^)	*R* ^2^	*n*_F_	*K*_F_ (mg^1−n^_F_L^nF^·g^−1^)	*R* ^2^
Cd	2	18.7	0.004	0.952	0.608	0.303	0.904
	4	10.9	0.006	0.925	0.601	0.228	0.877
	8	5.65	0.007	0.952	0.565	0.148	0.911
Cu	2	57.5	0.0458	0.965	0.330	9.40	0.996
	4	50.1	0.0338	0.971	0.362	6.74	0.999
	8	38.6	0.0642	0.977	0.359	6.05	0.976
Cr	2	70.2	0.0347	0.997	0.358	9.57	0.954
	4	52.3	0.0273	0.982	0.489	3.85	0.948
	8	32.2	0.0179	0.991	0.531	1.71	0.987
MB	2	3.12	0.0285	0.989	0.408	0.351	0.986
	4	1.58	0.0332	0.978	0.522	0.121	0.940
	8	0.930	0.0387	0.930	0.618	0.0524	0.927

## Data Availability

The data supporting the findings of this study are available within the article.
